# Plasma-Based microRNA Expression Analysis in Advanced Stage NSCLC Patients Treated with Nivolumab

**DOI:** 10.3390/cancers14194739

**Published:** 2022-09-28

**Authors:** Alexia Monastirioti, Chara Papadaki, Despoina Kalapanida, Konstantinos Rounis, Kleita Michaelidou, Maria A. Papadaki, Dimitrios Mavroudis, Sofia Agelaki

**Affiliations:** 1 Laboratory of Translational Oncology, School of Medicine, University of Crete, Vassilika Vouton, 71003 Heraklion, Crete, Greece; 2 Department of Medical Oncology, University General Hospital of Heraklion, Vassilika Vouton, 71110 Heraklion, Crete, Greece; 3 Thoracic Oncology Center, Theme Cancer, Karolinska University Hospital, 17164 Stockholm, Sweden; 4 Department of Oncology-Pathology, Karolinska Institutet, 17164 Stockholm, Sweden

**Keywords:** circulating miRNAs, miRNAs, NSCLC, immune checkpoint inhibitors, immunotherapy, immune response, survival, Nivolumab, PD-1

## Abstract

**Simple Summary:**

Nivolumab (anti-PD-1 inhibitor) is the first monoclonal antibody approved for the treatment of NSCLC, with research results showing that patients who had received previous lines of therapy had a better response to this treatment and better overall survival. Tissue-level analyses fail to capture the dynamic tumor-host relationship, in contrast to circulating biomarkers, which can reflect the systemic response of the tumor, allowing for repeated sampling and monitoring. In the context of liquid biopsy, microRNAs are studied as biomarkers of immunotherapy efficacy based on their role in regulating antitumor immunity. The present study suggests that miR-200c and miR-34a plasma expression levels have a prognostic role in patients with advanced NSCLC receiving Nivolumab. It further supports that the expression profile of circulating immunomodulatory microRNAs provides information on the survival of patients with advanced NSCLC receiving Nivolumab and could represent promising circulating biomarkers that may provide information about patients’ responses to immunotherapy.

**Abstract:**

Since circulating microRNAs (miRNAs) are involved in the modulation of the immune response, they are tested as liquid biopsy-based biomarkers in patients with NSCLC treated with immunotherapy. We analyzed the expression levels and examined the clinical significance of immunoregulatory miRNAs involved in immune checkpoint regulation (miR-34a, miR-200b, miR-200c), T-cell activity (miR-155), and the function of myeloid-derived suppressive cells (MDSCs) (miR-223) or regulatory T lymphocytes (Tregs) (miR-146a), in patients with advanced NSCLC (N = 69) treated with anti-PD-1 (Nivolumab) immunotherapy as 2nd or 3rd line of treatment therapy. Plasma levels of circulating miRNAs were analyzed by RT-qPCR before the initiation of immunotherapy. Expression of miR-34a, miR-146a, mir-200c, and miR-223 was found to be associated with response to immunotherapy. High miR-200c expression emerged as an independent prognostic factor for inferior overall survival in all patients with NSCLC (OS, HR: 2.243, 95% CI: 1.208–4.163; *p* = 0.010) and in patients with non-Squamous (non-SqCC) subtype (N = 38) (HR: 2.809, 95% CI: 1.116–7.074; *p* = 0.028). Low miR-34a expression independently predicted for shorter OS (HR: 3.189, 95% CI: 1.193–8.527; *p* = 0.021) in the non-SqCC subgroup. Our findings suggest that alterations in circulating miR-200c and miR-34a expression levels are associated with the response and outcome in patients with advanced NSCLC treated with anti-PD1 immunotherapy.

## 1. Introduction

Despite the evolution of therapeutic options, advanced NSCLC remains incurable [1], and approximately only 5% of the patients exceed the 5-year survival rates [2]. The advent of immune checkpoint inhibitors (ICIs) targeting components of the immune system responsible for immune evasion by the tumor cells has led to prolonged progression-free survival (PFS) and overall survival (OS) [3].

ICIs are monoclonal antibodies that aim the immune system activation by inhibiting several targets that suppress T-cell function and proliferation [4]. Over the years, the FDA has approved a variety of drugs against tumors, including NSCLC, which have exhibited promising results [5]. The first approved ICI for patients with advanced NSCLC was Nivolumab, a human IgG4 anti-PD-1 monoclonal antibody that blocks the interaction of PD-1 with PD-L1/PD-L2 [6]. CheckMate 017 and CheckMate 057 (phase III clinical trials) enrolled heavily pretreated stage III/IV patients with squamous-cell NSCLC and non-squamous-cell NSCLC, exhibiting disease progression during or after platinum-based chemotherapy, respectively. Patients treated with Nivolumab were reported to have longer OS compared to those treated with docetaxel in both studies [5,7]. Furthermore, the survival benefit of Nivolumab lasted over 2 years, regardless of the patient’s histology type [8]. Last year, Borghaei et al. published a 5-year follow-up report of these two phase III clinical trials, where “Nivolumab continued to demonstrate survival benefits over docetaxel, by exhibiting an increase in the OS rate” [9].

Despite these significant results, the benefit of immunotherapy is limited to only a subset of patients, while most patients eventually develop resistance to immunotherapy [10]. Therefore, the identification of patients that will have lasting clinical benefits is crucial [10]. Currently, PD-L1 quantification in tumor biopsies is the most widely validated and accepted biomarker in the selection of patients who are more likely to benefit from immunotherapy [11,12]. Also, other predictive factors associated with response to ICIs have been studied, such as high tumor mutational burden, DNA repair defects, and the presence of tumor-specific neo-antigens [11]. Yet, their predictive and prognostic role is still debated [1] because they do not reflect the tumor heterogeneity, as well as the dynamic interactions over time in the tumor microenvironment (TME) [12]. In this regard, characterization of the TME, along with the subsets of immune cells that have infiltrated it, may aid in the identification of factors that are favorable or undesirable for patients [13,14].

Over the past years, there has been an increasing interest in the field of liquid biopsy, and data resulting from studies are pointing to miRNAs as promising biomarkers for immunotherapy efficacy based on their role as regulators of antitumor immune responses [15]. MiRNAs may influence the course of tumor progression since they are central mediators of the interactions between the tumor cells and the immune cells in the TME [16]. Boeri et al., in their study, resulted in a panel of miRNAs that could identify patients with advanced NSCLC with worse response to immunotherapy [1], while Fan et al. identified another panel of miRNAs that could predict patients with better response to immunotherapy treatment [17].

In our study, we hypothesized that circulating miRNAs participating in the regulation of immune checkpoints, Tregs and MDSCs may have prognostic implications as biomarkers in patients with advanced NSCLC treated with second-line anti-PD-1 (Nivolumab). Specifically, miR-34a has been shown to suppress the expression of PD-L1 as a downstream regulator of p53 [18]. MiR-200b and miR-200c (part of the miR-200 family) through the miR-200/ZEB1 axis negatively regulate the EMT process and metastasis by targeting PD-L1 [19]. MiR-146a [20] and miR-155 [21] are critical regulators of Tregs development and suppressive functions, and miR-223 has been found to participate in myeloid cell proliferation and differentiation [22].

## 2. Materials and Methods

### 2.1. Patients’ Characteristics, Healthy Volunteers, and Blood Sample Collection

**Figure 1:**
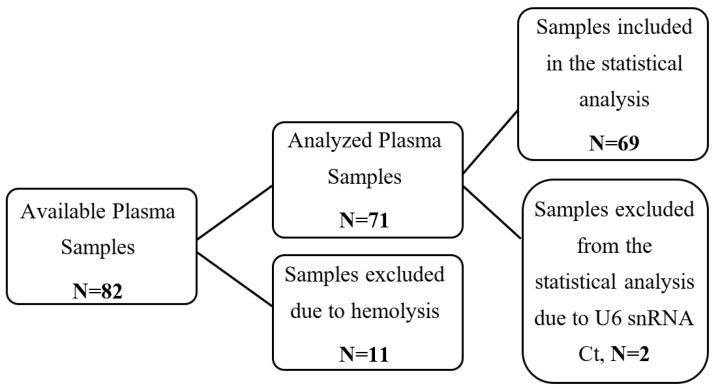
Flow Diagram for study participants treated with Nivolumab monotherapy.

Patients with advanced (not amenable to radical loco-regional treatment) or metastatic NSCLC, treated with 2nd- or 3rd-line Nivolumab (recurrence or progression after first-line chemotherapy) at the Department of Medical Oncology, University General Hospital of Heraklion, Crete, Greece, from 2018 to 2020, were enrolled in the present study. The study was conducted according to the guidelines of the Declaration of Helsinki and approved by the Ethics and Scientific Committee of the University General Hospital of Heraklion, Crete, Greece (ID: 13725/8-11-17, Crete, Greece). A total of 82 patients with available plasma samples prior to the initiation of immunotherapy were retrieved (Figure 1). Hemolyzed plasma samples (N = 11) were excluded from the study (Figure 1). Clinical and follow-up data of the patients were collected prospectively.

For the normalization of the miRNAs’ expression values, blood samples of 33 healthy volunteers were used as a control group. These samples were obtained as part of the volunteer blood donation procedure in the Blood Bank Department of the University General Hospital of Heraklion, Crete, Greece. Healthy volunteers’ median age was 63 years, 30 of whom were male and 3 females. All patients and volunteers have signed an informed consent form for their participation in the current research program before their plasma sample collection. Peripheral blood from patients and healthy volunteers was collected in ethylenediaminetetraacetic acid (EDTA) tubes. Plasma was isolated from whole blood by centrifugation and stored at −80 °C until further use.

### 2.2. miRNA Expression: RNA Isolation from Plasma Samples & Quantitative Real-Time PCR Analysis

Total RNA was extracted from 400 μL of plasma using Trizol-LS (Ambion, Life Technologies) according to the manufacturer’s instructions, and synthetic miRNA from *C. elegans* (Cel-miR-39, Qiagen Inc., Hilden, Germany) was used as an exogenous control to allow for normalization of sample-to-sample variations, as previously described [23]. Chloroform was also added to each sample to allow for aqueous phase separation. The RNA pellet was resuspended in 50 μL RNAse-free water, and the samples were stored at −80 °C until further use in the subsequent real-time qPCR.

Reverse Transcription and RT-qPCR were performed according to the manufacturer’s instructions, as previously described [23]. Total RNA was reversed transcribed using TaqMan miRNA Reverse Transcription kit and miRNA-specific stem-loop primers (Table S1: miRNA assay IDs, Applied Biosystems, Foster City, CA, USA). The reverse transcription reaction was performed in a final volume of 5 μL, and the complementary DNA (cDNA) was later diluted at 20 μL final volume. The RT-qPCR reaction was carried out on a ViiA 7 Real-Time PCR System (Applied Biosystems, Foster City, CA, USA). All the assays were performed in triplicates. Appropriate negative controls were used in both cDNA synthesis, and RT-qPCR reactions, where RNA input was replaced by H2O and no template control was used, respectively [23]. The average expression levels for each miRNA were calculated by the 2−ΔCt method relative to the average of U6 snRNA.

U6 snRNA was chosen as a suitable reference gene for normalization due to expression stability and reproducibility among the group of patients and the group of healthy volunteers (Mann-Whitney test, *p* = 0.160, Figure S1). Acceptable mean cycle threshold (Ct) range were for U6 snRNA: 30 < Ct U6 < 33 and for cel-miR-39: 20 < Ct cel-miR-39 < 22. Samples with mean Ct outside of these ranges were excluded from the analysis (N = 2 for U6 snRNA and N = 0 for cel-miR-39). The fold change in target miRNAs relative to their expression in healthy volunteers was determined using the 2^−ΔΔCt^ method [24]. Median Ct values, SD, and median miRNA expression values for both patients and healthy volunteers are depicted in Table S2.

### 2.3. Assessment of Outcome of Immunotherapy

Computed tomography (CT) and magnetic resonance imaging (MRI) scans were used to assess the patients’ responses to treatment based on the Response Evaluation Criteria in Solid Tumors (RECIST) 1.1 criteria [25]. Based on the response to immunotherapy, patients were characterized as having a partial response (PR), stable disease (SD), or progressive disease (PD). Objective response rate (ORR) included the patients that achieved PR as the best response to therapy, disease control rate (DCR) included the patients that achieved PR or SD as the best response to therapy and prolonged duration of disease control (PDDC) included patients that achieved PR or SD and did not exhibit disease progression for a period of 6 months or longer. Progression-free survival (PFS) and overall survival (OS) were measured from the initiation of 2nd or 3rd line immunotherapy treatment until the first documentation of disease progression (or death) and death, respectively. The date of the last follow-up was used in patients that had no disease progression and were still alive at the time of data analysis.

### 2.4. Statistical Analysis 

SPSS software package version 22.0 (accessed on 20 September 2021; statistical package of the social sciences, SPSS Inc., Chicago, IL, USA) was used to implement the statistical analysis. The miRNAs’ relative expression values do not follow a normal distribution (*p* < 0.05, Kolmogorov-Smirnov and Shapiro-Wilk tests). Based on the median value of each miRNA, patients with expression values higher than or equal to the median values were classified as high expression, whereas those with expression values lower than the median values were classified as low expression. The correlation of miRNA expression with clinicopathological characteristics was assessed using the Mann-Whitney and chi-square tests. The association of miRNAs expression levels with PFS and OS was assessed using the Kaplan–Meier method, log-rank test (Mantel–Cox), and Cox proportional hazard regression models. Parameters with statistical significance in the univariate analysis were used to compose the multivariate Cox regression analysis. Statistical significance was set at *p* < 0.05 (two-sided test). This report is written based on the Reporting Recommendations for Tumor Marker Prognostic Studies (REMARK criteria) [26].

## 3. Results

### 3.1. Study Design and Patients’ Clinicopathological Characteristics

The flow of the present study and the clinicopathological characteristics are summarized in Figure 1 and Table 1, respectively. A total of 69 patients treated with Nivolumab monotherapy as second- or third-line therapy were included in this study and analyzed. The median age was 70.5 years (range: 39–82), 84.1% of the patients were male, and 15.9% were female. Of the total, 55.1% had non-SqCC histologic subtype, and 44.9% had SqCC. Regarding patients’ response to immunotherapy treatment, 11.6% experienced PR (partial response), 40.6% SD (stable disease) and 47.8% PD (progressive disease) based on the RECIST 1.1 criteria [25].

### 3.2. miRNA Expression and Patients’ Clinicopathological Characteristics

The relative expression of miR-34a (Mann-Whitney test; *p* < 0.001), miR-146a (Mann-Whitney test; *p* < 0.001), miR-200b (Mann-Whitney test; *p* < 0.001), miR-200c (Mann-Whitney test; *p* < 0.001) and miR-233 (Mann-Whitney test; *p* < 0.001), were found to be higher in patients with NSCLC (N = 69) compared to healthy volunteers (N = 33) (Figure S2).

Regarding miRNA expression and patients’ clinicopathological characteristics, expression of miR-200b and miR-200c was found to be differentially expressed in the histologic subtypes, and both their expression was higher in patients with non-SqCC subtype compared to SqCC (Mann-Whitney test, *p* = 0.001, and *p* = 0.019, respectively, Figure S3A). High expression of miR-200b and miR-200c was correlated with bone metastases in all patients (Mann-Whitney test, *p* = 0.002 and *p* = 0.020, respectively, Figure S3B) and in patients with SqCC subtype (Mann-Whitney test, *p* = 0.028 and *p* = 0.019, respectively, Figure S3B). Additionally, high expression of miR-34a, miR-200b and miR-200c were associated with liver metastases (Mann-Whitney test, *p* = 0.042, *p* = 0.018 and *p* = 0.006, respectively, Figure S3C) in all patients and high miR-200c expression was also associated with liver metastases in patients with non-SqCC subtype (Mann-Whitney test, *p* = 0.048, Figure S3C). Furthermore, higher expression of miR-200b and miR-200c was correlated with the presence of multiple metastatic sites (>3) (Mann-Whitney test, *p* = 0.013 and *p* = 0.022, respectively, Figure S3D). No other correlations were observed comparing miRNA expression with patients’ clinicopathological characteristics.

### 3.3. miRNA Expression and Response to Immunotherapy

Regarding miRNA expression and response to treatment, there was no association in all patients. However, in patients with the SqCC subtype, miR-146a and miR-223 were found to be correlated with PDDC, while in patients with the non-SqCC subtype, miR-34a was correlated with DCR and miR-200c with ORR and PDDC (Table 2). More specifically in the SqCC subgroup, patients with low miR-146a and low miR-223 expressions exhibited shorter duration of disease control (88.9% SD < 6 months/PD vs. 11.1% PR/SD > 6 months, *p* = 0.014 and 88.2% SD < 6 months/PD vs. 11.8% PR/SD > 6 months, *p* = 0.026, respectively, chi-square test).

Additionally, in the non-SqCC subgroup, 75.0% of patients with low miR-34a expression exhibited PD, compared to 25.0% of patients with low miR-34a expression who exhibited PR/SD, while 95.0% of patients with high miR-200c expression exhibited SD/PD, compared to 5.0% of patients with high miR-200c expression who exhibited PR (*p* = 0.038 and *p* = 0.049, respectively, chi-square test). Lastly, patients with high miR-200c expression exhibited shorter duration of disease control (80.0% SD < 6 months/PD vs. 20.0% PR/SD > 6 months, *p* = 0.028, chi-square test). Statistical correlation of miRNAs expression levels and response to immunotherapy exhibited in the following table are depicted as box plots in the Supplementary (Figure S4).

### 3.4. miRNA Expression and Survival Outcomes

**Figure 2:**
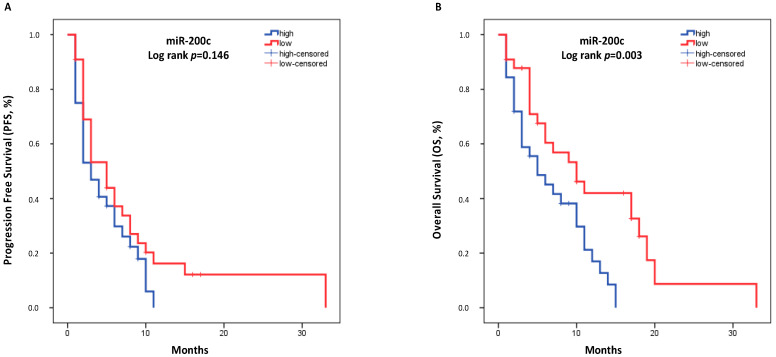
Kaplan–Meier analysis for PFS (**A**) and OS (**B**) based on the miR-200c expression levels in the plasma of patients with advanced NSCLC (N = 69). Median expression values classified patients into high and low expression groups. Curves were compared using the log-rank test. *p*-values are shown.

In all patients (N = 69), the median PFS was 4.0 months (95% CI: 2.180–5.820), and the median OS was 8.0 months (95% CI: 4.758–11.242). There was no correlation between miRNA expression levels and PFS (Figure S5). However, patients with high miR-200c expression had shorter OS (high expression: 5.0 months vs. low expression: 10.0 months, *p* = 0.003, Figure 2). The expression of the remaining miRNAs was not associated with OS (Figure S5).

The Cox regression model showed that there were no statistically significant correlations among miRNAs’ expression and clinicopathological characteristics with PFS (Table 3).

Univariate Cox regression analysis (N = 69) revealed that high miR-200c was associated with shorter OS (HR: 2.382, 95% CI: 1.291–4.393; *p* = 0.005) (Table 3). Among the other factors, only PS ≥ 2 was associated with shorter OS (HR: 2.295, 95% CI: 1.223–4.305; *p* = 0.010) (Table 3). In the multivariate Cox regression analysis (N = 69), high miR-200c expression emerged as an independent prognostic factor for worse OS (HR: 2.243, 95% CI: 1.208–4.163; *p* = 0.010; Table 3).

### 3.5. miRNA Expression and Survival Outcomes in the Histologic Subgroups

As mentioned in Section 3.1, 44.9% of patients had the SqCC histologic subtype (N = 31), 55.1% had the non-SqCC histologic subtype (N = 38), and the characteristics for each group of patients are depicted and summarized in Table 1. In patients with the SqCC subtype, the median PFS was 3.0 months (95% CI: 1.226–4.774), and the median OS was 6.0 months (95% CI: 0.791–11.209). There was no association of miRNAs expression levels with neither PFS nor OS in this subgroup of patients (Figure S6). Through the Univariate Cox regression analysis (N = 31), there was no association with PFS; however, only PS ≥ 2 was associated with shorter OS (HR: 4.267, 95% CI: 1.584–11.494; *p* = 0.004; Table S3).

**Figure 3:**
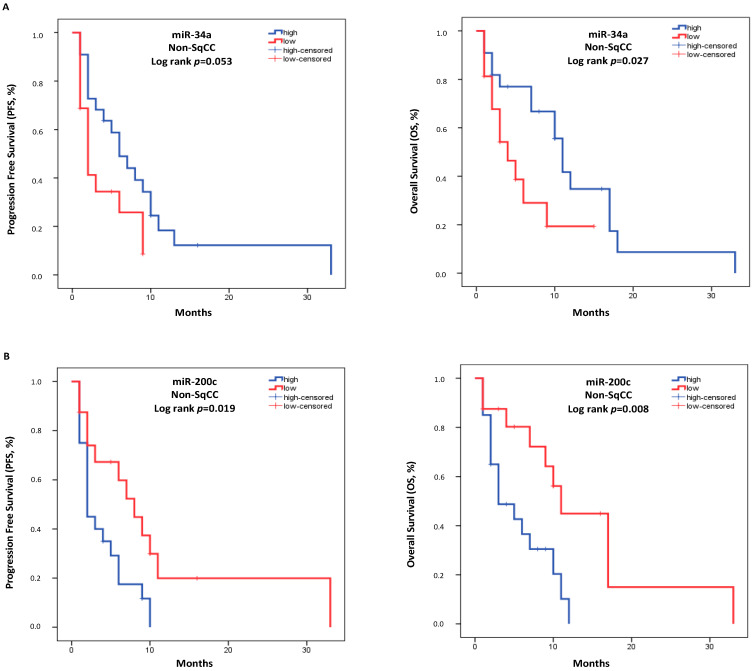
Kaplan–Meier analysis for PFS (Left) and OS (Right) based on the miR-34a (**A**) and miR-200c (**B**) expression levels in the plasma of patients with non-SqCC subtype (N = 38). Median expression values classified patients into high and low expression groups. Curves were compared using the log-rank test. *p*-values are shown.

In patients with the non-SqCC subtype, the median PFS was 5.0 months (95% CI: 1.799–8.201), and the median OS was 9.0 months (95% CI: 5.091–12.909). Low miR-34a expression was associated with shorter OS (high expression: 11.0 months vs. low expression: 4.0 months, *p* = 0.027, Figure 3A). Additionally, high miR-200c expression was associated with both shorter PFS and OS (high expression: 2.0 months vs. low expression: 8.0 months, *p* = 0.019 and high expression: 3.0 months vs. low expression: 11.0 months, *p* = 0.008, respectively, Figure 3B). There was no other correlation with PFS or OS for the remaining miRNAs in this group of patients (Figure S7).

Through the Univariate Cox Regression analysis (N = 38), high miR-200c expression was associated with shorter PFS (HR: 2.346, 95% CI: 1.053–5.225; *p* = 0.037). However, there were no other statistically significant correlations with PFS (Table 4). In addition, low miR-34a expression and high miR-200c expression were associated with shorter OS (HR: 2.500, 95% CI: 1.050–5.952; *p* = 0.038 and HR: 3.112, 95% CI: 1.247–7.766; *p* = 0.015, respectively; Table 4). In the multivariate Cox regression analysis (N = 38), both low miR-34a expression and high miR-200c expression emerged as independent prognostic factors for worse OS (HR: 3.189, 95% CI: 1.193–8.527; *p* = 0.021 and HR: 2.809, 95% CI: 1.116–7.074; *p* = 0.028, respectively; Table 4).

## 4. Discussion

MiRNAs expression has been documented to be dysregulated in tumors, forming a particular expression profile, and this dysregulation has been liked to their origin, disease progression, responses to therapy, and survival rates of cancer patients [27]. The original hypothesis of the present study was to examine the role of immune-related miRNAs in the plasma of NSCLC patients treated with Nivolumab. Following a thorough literature review, we concluded by examining miRNAs with a reported role in immune regulation and, more specifically, miRNAs that participate in the regulation of immune checkpoints (miR-34a, miR-200b, miR-200c), T-cell activity (miR-155), Tregs (miR-146a) and the function of myeloid-derived suppressive cells (MDSCs) (miR-223). These miRNAs were chosen based on their already proposed role in cancer immunity and their correlation with survival measures and overall response in cancer patients. The aim of our study was to investigate whether immune-related miRNAs hold any predictive significance when assessed in the plasma of NSCLC patients. MiRNAs are subjected to molecular changes posed by the tumor cells, and as a result, circulating miRNAs could be considered reliable disease monitoring markers [27]. Expression levels of circulating miR-34a, miR-146a, miR-155, miR-200b, miR-200c, and miR-223 were assessed in the plasma of patients with advanced NSCLC treated with second- or third-line anti-PD1 therapy and were correlated with survival outcomes. High miR-200c expression emerged as an independent prognostic factor for poor OS, both in all patients and those with the non-SqCC subtype. Additionally, low miR-34a expression independently predicted worse OS in patients with non-SqCC subtype. Furthermore, in patients with the SqCC subtype, both low miR-146a and miR-223 expressions were associated with PDDC, whereas in patients with the non-SqCC subtype, high miR-200c expression was correlated with ORR and PDDC, and low miR-34a expression with DCR.

Mir-200c is one of the most studied miRNAs regarding its role in tumor progression. The miR-200 family members (miR-200a, -200b, -200c, -141, -429) directly targets the zinc-finger E-box-binding homeobox 1 (ZEB1), a transcription activator of EMT, resulting in inhibition of metastasis and invasion of tumor cells [19]. Additionally, PD-L1 is a downstream target of the miR-200/ZEB1 axis, known to facilitate immunosuppression in the tumor milieu. PD-L1 expression from tumor cells promotes CD8+ TILs exhaustion, thus supporting tumor growth and metastasis [19]. In this context, miR-200c targets the 3′ untranslated region (UTR) of PD-L1 and inhibits its expression, along with reversing the CD8+ T cells exhaustion. In early-stage NSCLC, low miR-200c expression levels have been associated with higher PD-L1 levels and higher EMT scores, resulting in unfavorable clinical outcomes [19]. However, there has been evidence for its function in tumor angiogenesis [28]. In vitro and functional studies have produced incompatible results regarding whether miR-200c acts as a tumor suppressor or promotes metastasis in different samples and cancer types [29,30,31].

In our results, high miR-200c expression was strongly associated with worse OS in all patients and, through the multivariate Cox model, independently predicted shorter OS. Additionally, in patients with non-SqCC subtype, high miR-200c expression was correlated with shorter PFS and OS. Through the univariate Cox model, high miR-200c was related to shorter PFS in patients with non-SqCC subtype. Also, through the multivariate Cox model, high miR-200c expression emerged as a negative independent predictor for OS in the non-SqCC subgroup. Moreover, higher miR-200c expression was associated with worse responses to therapy in patients with non-SqCC subtype.

In accordance with our results, miR-200c expression was higher in tumor tissue samples from patients with NSCLC compared to non-tumor samples, and its expression was associated with shorter survival [32,33]. Furthermore, high miR-200c expression in resected NSCLC tissue samples was associated with worse OS in all patients and in patients with the non-SqCC subtype of the cohort’s patients [34]. Earlier this year, in ovarian cancer cell lines, higher miR-200c expression was associated with a less tumorigenic microenvironment and inhibited PD-L1 expression [35]. Contrary to these results, another group studying also ovarian cancer found that in tissue samples, overexpression of miR-200c was correlated with a more aggressive tumor [36]. Taken together findings from previous studies and the results of our study, we have come to believe that miR-200c is differentially expressed in the tumor microenvironment and in circulation. Given the heterogeneity of the tumor regarding NSCLC, further studies are essential to understand the role of miR-200c, and mapping its origin might provide a deeper understanding of its functions.

MiR-34a is one of the most studied, highly conserved miRNAs with tumor suppressive functions [37]. MiR-34a expression levels are suggested to be closely linked with multiple components of the immune system and tumor immunity, such as NK cell activity, the polarization of macrophages, differentiation of MDSCs, and T-cell populations’ activation and proliferation [38]. Additionally, miR-34a has been found to participate in the regulation of the metabolism of tumor cells and is able to rewire their growth abilities, proliferation, and maintenance [38]. Furthermore, miR-34a negatively regulates the EMT process by targeting a variety of key genes and can possibly reverse this process [38]. Given its tumor suppressive functions, miR-34a is often downregulated in solid tumors. MiR-34a directly targets the 3′ UTR region of PD-L1 and suppresses its expression [39]. In NSCLC, low miR-34a expression levels are associated with overexpression of PD-L1 on the cell surface of tumor cells [18]. As part of the p53/miR-34/PD-L1 axis, in NSCLC, low miR-34a expression and high PD-L1 expression have been associated with poor clinical outcomes [18]. In patients with NSCLC with malignant pleural effusion, highly tumorigenic CD44-high cell subsets were presented with low miR-34a expression levels, and miR-34a-mediated expression inhibited CD44-high cell colony formation [40].

In our results, in patients with non-SqCC subtype, low miR-34a expression was correlated with poor PFS and OS and, through the multivariate Cox model, independently predicted worse OS. These findings are supported by previous studies, where high miR-34a expression in the plasma of patients with NSCLC has been correlated with better PFS and OS [41], while in women with triple-negative breast cancer (TNBC), low circulating miR-34a expression was associated with worse OS [42]. Moreover, tissue analysis of surgically resected samples of patients with NSCLC showed that low expression levels of miR-34a were correlated with a higher probability of relapse [43]. Also, in the present study, high miR-34a expression was associated with better responses to anti-PD-1 regimens in patients with non-SqCC subtype, which is supported by our previous work, in which case lower miR-34a expression levels were observed in patients with poor response to second- and third-line immunotherapy [44].

MiR-223, a key regulator of myeloid differentiation, is highly expressed during hematopoiesis and negatively regulates MDSCs [45]. It has also been characterized as a central mediator of the immune response during inflammation by modulating granulocyte differentiation, macrophage polarization, dendritic cell differentiation, T-cell mediated inflammation, and endothelial and epithelial inflammation [46]. MiR-223 has been described as a tumor suppressor in a variety of tumors [47] and has been found to suppress cell growth and proliferation and inhibit colony formation in HeLa cell lines [46]. In the TME, deregulation of miR-223 often leads to MDSCs expansion and activation [45]. In lung cancer, miR-223 was found to be downregulated and participate in cell cycle regulation, while its upregulation in vitro resulted in inhibition of tumor cell migration and invasion [48]. D’Antona et al., in their study, validated miR-223 as a reproducible and effective serum biomarker for patients with early NSCLC [49].

In previous studies, miR-223 was found to be downregulated in squamous lung cancer tissues compared to adjacent normal tissues. In in vitro experiments of the same study, miR-223 was also found to inhibit proliferation and migration, while in in vivo experiments, it suppressed tumor growth [50]. Additionally, circulating levels of miR-223 in patients with osteosarcoma were downregulated, and shorter survival was correlated with low miR-223 expression [51], and in TNBC tissue samples, high miR-223 expression was associated with improved survival measures [52]. The results of our study are supported by these findings. Specifically, we found that in the SqCC subgroup of patients, low miR-223 expression was negatively associated with prolonged disease control, and these patients exhibited disease progression earlier than the patients with high miR-223 expression.

MiR-146a has been characterized as a central regulator of innate immune responses [53], with a distinct role in controlling various biological functions of antigen-presenting cells [54]. More specifically, studies have suggested the role of miR-146a as a mediator of dendritic cell survival and activation and post-inflammatory signaling [55]. In cancer, miR-146a has been implicated in the development and inhibition of tumor progression [56]. In esophageal cancer, miR-146a was downregulated in tumor tissue samples compared to normal tissue, and this was correlated with worse PFS and OS [57]. Similarly, in ovarian cancer, low miR-146a expression was associated with poorer PFS and OS [58].

In NSCLC, mir-146a has been found to act both as an oncomiR and as a tumor suppressor. However, most studies have proposed a tumor suppressive role of miR-146a. MiR-146a is reported to be downregulated in NSCLC cell lines and tissue samples [59] and, as such, has been studied as a potential biomarker for lung cancer detection [60]. Additionally, in tumor samples from patients with NSCLC, low miR-146a expression levels were associated with advanced tumor stage, metastases, and shorter PFS compared to patients with high miR-146a expression [59]. Our results further support these studies. In detail, we found that patients with low miR-146a expression exhibited progressive disease at a shorter period, thus proposing a tumor suppressive role of this miRNA.

In the present study, pre-analytical and analytical parameters were considered thoroughly, considering the variables that could lead to bias in miRNA quantification [61,62]. Presently, studies regarding the correlation of miRNAs with the outcome of NSCLC patients undergoing immunotherapy treatment are limited. As a result, available data on miRNAs to drive any significant conclusion on their role in response prediction and survival are finite, mostly due to the small size of patients enrolled in those studies and the different techniques [63,64]. On the contrary, results derived from our study are based on a rather large number of patients (n = 69). Also, the significance of our study lies in the correlation of the expression profile of immunomodulatory miRNAs with the survival, the response to immunotherapy, and the clinicopathological characteristics of the patients and not as a tool of NSCLC detection in clinical practice. Furthermore, it should also be stressed that the function of the circulating miRNAs in immune response remains unclear as yet, and further investigation is required to unravel their biological function in circulation.

The external validation of our results on heterogeneous and independent datasets is among our future goals to strengthen the significance of our findings for patients with NSCLC treated with anti-PD-1 immunotherapy regimens. Additionally, we aim to enroll more patients in our future goals to further validate the prognostic/predictive role of these miRNAs. One other limitation of the present study is the lack of experimental results from later time points during the therapy of our patients to analyze possible variations of miRNA expression patterns. However, our future goal is to analyze the expression levels of these miRNAs in the mononuclear cells of the peripheral blood (PBMCs) of this cohort of patients, with a view to understanding the origin of the miRNAs, along with investigating the role of these miRNAs in a cohort of patients receiving first-line immunotherapy.

## 5. Conclusions

MiRNAs are stably expressed in numerous body fluids, yet the association of circulating miRNAs with miRNAs expressed in tissues remains vague. Furthermore, it should also be mentioned that the function of the circulating miRNAs in the modulation of immune response remains unclear as yet, and further investigation is required to unravel their biological function in circulation. Nevertheless, the interpretation of the role of circulating miRNAs as potential biomarkers is of great importance [65]. The tumor-originated alterations of circulating miRNAs in cancer patients represent a promising, non-invasive tool with prognostic implications. These small non-coding RNAs released into the circulation by the tumor carry important information regarding the origin, development, and progression of the tumor, which reflects their prognostic potential [66].

To summarize, our results further support the hypothesis that the expression profile of miRNAs, involved in the regulation of key components of the immune system, is associated with survival measures in patients with advanced NSCLC treated with anti-PD-1 regimens. To achieve a more comprehensive understanding of the regulatory interactions of the immune checkpoints and the immune components, future research should be directed toward the origin of circulating miRNAs. These studies will provide insight and further clarify the significance of liquid biopsy in the treatment of NSCLC.

## Figures and Tables

**Table 1:** Clinicopathological Characteristics of Patients.

	All Patients		SqCC		Non-SqCC		
Characteristic	N	%	N	%	N	%	*p* Value
Number of patients	69		31	44.9	38	55.1	
Gender							0.009 ^a^
Male	58	84.1	30	96.8	28	73.7	
Female	11	15.9	1	3.2	10	26.3	
Age (years)							0.050 ^a^
median (range)	70.5 (39–82)		72 (55–81)		69 (39–82)		
ECOG PS							0.553 ^a^
0	24	34.8	9	29.0	15	39.5	
1	31	44.9	16	51.6	15	39.5	
2	14	20.2	6	19.3	8	21.1	
Smoking Status							0.052
Current Smoker	42	60.9	15	48.4	27	71.1	
Former Smoker	23	33.3	15	48.4	8	21.1	
Never Smoker	4	5.8	1	3.2	3	7.9	
Histology							ns ^a^
Non-SqCC	38	55.1					
Squamous	31	44.9					
Number of metastatic sites							0.217 ^a^
0	2	2.9	1	3.2	1	2.6	
1	16	23.1	9	29.0	7	18.4	
2	31	44.9	17	54.8	14	36.8	
≥3	20	29.0	4	12.9	16	42.1	
Line of Immunotherapy Treatment							0.083
2nd line	59	85.5	29	93.5	30	78.9	
3rd line	10	14.5	2	6.5	8	21.1	
Response							0.454 ^a^
PR	8	11.6	2	6.5	6	15.8	
SD	28	40.6	14	45.2	14	36.8	
PD	33	47.8	15	48.4	18	47.4	

ECOG PS, Eastern Cooperative Oncology Group Performance Status; PR, partial response; SD, stable disease; PD, progressive disease; ^a^ Pearson’s chi-squared test for comparison between patients with SC and non-SQCC; number of metastatic sites depicts the number of affected organs.

**Table 2:** MiRNAs Correlation and Response to Treatment.

Response		ORR						DCR						PDDC					
Histology		SqCC			Non-SqCC			SqCC			Non-SqCC			SqCC			Non-SqCC		
Expression Levels		PR (%)	SD + PD (%)	*p* Value	PR (%)	SD + PD (%)	*p* Value	PR + SD (%)	PD (%)	*p* Value	PR + SD (%)	PD (%)	*p* Value	PR + SD > 6 Months (%)	SD < 6 Months + PD (%)	*p* Value	PR + SD > 6 Months (%)	SD < 6 Months + PD (%)	*p* Value
miR-34a	High	7.1	92.9	0.708	22.7	77.3	0.180	50.0	50.0	0.578	59.1	40.9	0.038 *	28.6	71.4	0.637	50.0	50.0	0.111
	Low	5.9	94.1		6.2	93.8		47.1	52.9		25.0	75.0		29.4	70.6		25.0	75.0	
miR-146a	High	7.7	92.3	0.671	13.0	87.0	0.444	61.5	38.5	0.189	39.1	60.9	0.299	53.8	46.2	0.014 *	30.4	69.6	0.142
	Low	5.6	94.4		20.0	80.0		38.9	61.1		53.3	46.7		11.1	88.9		53.3	46.7	
miR-155	High	7.1	92.9	0.708	22.7	77.3	0.180	64.3	35.7	0.106	45.5	54.5	0.590	42.9	57.1	0.127	36.4	63.6	0.449
	Low	5.9	94.1		6.2	93.8		35.3	64.7		43.8	56.2		17.6	82.4		43.8	56.2	
miR-200b	High	9.1	90.9	0.591	20.8	79.2	0.264	54.5	45.5	0.447	41.7	58.3	0.435	45.5	54.5	0.140	37.5	62.5	0.505
	Low	5.0	95.0		7.1	92.9		45.0	55.0		50.0	50.0		20.0	80.0		42.9	57.1	
mir-200c	High	8.3	91.7	0.665	5.0	95.0	0.049 *	58.3	41.7	0.413	30.0	70.0	0.106	41.7	58.3	0.263	20.0	80.0	0.028 *
	Low	5.9	94.1		31.2	68.8		47.1	52.9		56.2	43.8		23.5	76.5		56.2	43.8	
miR-223	High	7.1	92.9	0.708	13.6	86.4	0.502	57.1	42.9	0.300	45.5	54.5	0.590	50.0	50.0	0.026 *	36.4	63.6	0.449
	Low	5.9	94.1		18.8	81.2		41.2	58.8		43.8	56.2		11.8	88.2		43.8	56.2	

ORR: Objective Response Rate; DCR: Disease Control Rate; PDDC: Prolonged Duration Disease Control; PR: Partial Response; SD: Stable Disease; PD: Progressive Disease; SqCC: Squamous; Non-SqCC: non-Squamous; Patients classified into high and low expression groups according to the median value of each miRNA; * *p* < 0.05.

**Table 3:** Univariate and Multivariate Cox Regression Analysis for Progression Free Survival (PFS) and Overall Survival (OS) in Patients with Advanced NSCLC (N = 69) Treated with Nivolumab.

Variable	Progression Free Survival (PFS)				Overall Survival (OS)			
	Univariate Analysis		Multivariate Analysis		Univariate Analysis		Multivariate Analysis	
	HR (95% CI)	*p* Value	HR (95% CI)	*p* Value	HR (95% CI)	*p* Value	HR (95% CI)	*p* Value
Age (<60 vs. ≥60)	1.106 (0.558–2.192)	0.773	-	-	1.105 (0.518–2.356)	0.796	-	-
Gender (male vs. female)	1.149 (0.564–2.341)	0.702	-	-	1.361 (0.633–2.929)	0.430	-	-
Smoker (Yes vs. No)	1.679 (0.522–5.403)	0.384	-	-	2.446 (0.589–10.154)	0.218	-	-
**ECOG PS (≥2 vs. 0–1)**	1.804 (0.981–3.318)	0.058	-	-	**2.295 (1.223–4.305)**	0.010 *	1.819 (0.949–3.486)	0.071
Histology (SqCC vs. Non-SqCC)	1.197 (0.712–2.011)	0.497	-	-	1.010 (0.584–1.748)	0.971	-	-
Immunotherapy Line (2nd vs. 3rd)	1.060 (0.534–2.104)	0.868	-	-	1.030 (0.497–2.134)	0.936	-	-
No. of Metastatic Sites (≥3 vs. 0–2)	1.202 (0.679–2.125)	0.528	-	-	1.760 (0.971–3.189)	0.062	-	-
miR-34a (low vs. high)	1.424 (0.848–2.390)	0.181	-	-	1.083 (0.623–1.881)	0.778	-	-
miR-146a (high vs. low)	1.110 (0.662–1.861)	0.693	-	-	1.180 (0.678–2.052)	0.558	-	-
miR-155 (high vs. low)	1.039 (0.616–1.752)	0.885	-	-	1.379 (0.785–2.426)	0.264	-	-
miR-200b (high vs. low)	1.076 (0.642–1.803)	0.781	-	-	1.618 (0.952–2.828)	0.092	-	-
**miR-200c (high vs. low)**	1.438 (0.838–2.468)	0.187	-	-	**2.382 (1.291–4.393)**	0.005 *	**2.243 (1.208–4.163)**	0.010 *
miR-223 (high vs. low)	1.163 (0.693–1.952)	0.568	-	-	1.379 (0.788–2.412)	0.260	-	-

HR, Hazard Ratio; CI, Confidence Intervals; ECOG PS, Eastern Cooperative Oncology Group Performance Status; patients classified into high and low expression groups according to the median value of each miRNA; Cox regression, * *p* < 0.05.

**Table 4:** Univariate and Multivariate Cox Regression Analysis for Progression Free Survival (PFS) and Overall Survival (OS) in Patients with non-SqCC Subtype (N = 38) Treated with Nivolumab.

Variable	Progression Free Survival (PFS)				Overall Survival (OS)			
	Univariate Analysis		Multivariate Analysis		Univariate Analysis		Multivariate Analysis	
	HR (95% CI)	*p* Value	HR (95% CI)	*p* Value	HR (95% CI)	*p* Value	HR (95% CI)	*p* Value
Age (<60 vs. ≥60)	1.247 (0.555–2.806)	0.593	-	-	1.066 (0.444–2.557)	0.886	-	-
Gender (male vs. female)	1.114 (0.498–2.494)	0.792	-	-	1.261 (0.518–3.069)	0.610	-	-
Smoker (Yes vs. No)	2.094 (0.493–8.889)	0.316	-	-	5.059 (0.670–38-183)	0.116	-	-
ECOG PS (≥2 vs. 0–1)	1.876 (0.813–4.330)	0.141	-	-	1.663 (0.715–3.869)	0.237	-	-
Immunotherapy Line (2nd vs. 3rd)	1.029 (0.456–2.324)	0.945	-	-	1.316 (0.564–3.071)	0.526	-	-
No. of Metastatic Sites (≥3 vs. 0–2)	1.314 (0.636–2.712)	0.461	-	-	1.646 (0.749–3.615)	0.215	-	-
miR-34a (low vs. high)	1.970 (0.918–4.226)	0.082	-	-	**2.500 (1.050–5.952)**	0.038 *	**3.189 (1.193–8.527)**	0.021*
miR-146a (high vs. low)	1.286 (0.621–2.664)	0.499	-	-	1.423 (0.622–3.252)	0.403	-	-
miR-155 (high vs. low)	1.203 (0.583–2.482)	0.617	-	-	1.198 (0.543–2.643)	0.655	-	-
miR-200b (high vs. low)	1.061 (0.507–2.221)	0.874	-	-	1.642 (0.712–3.787)	0.245	-	-
miR-200c (high vs. low)	**2.346 (1.053–5.226)**	0.037*	-	-	**3.112 (1.247–7.766)**	0.015 *	**2.809 (1.116–7.074)**	0.028*
miR-223 (high vs. low)	1.245 (0.603–2.567)	0.554	-	-	1.759 (0.753–4.108)	0.192	-	-

HR, Hazard Ratio; CI, Confidence Intervals; ECOG PS, Eastern Cooperative Oncology Group Performance Status; patients classified into high and low expression groups according to the median value of each miRNA; Cox regression, * *p* < 0.05.

## Data Availability

Data will be available upon request from the corresponding author.

## Supplementary Materials

The following are available online at https://www.mdpi.com/article/10.3390/cancers14194739/s1, Table S1: Assay ID for each miRNA used in the study; Figure S1: U6 snRNA expression levels between patients with NSCLC and healthy donors. Mann-Whitney test was used to determine statistically significant differences, and the results were displayed on box plots. The horizontal line depicts the median Ct value, whereas the length of the boxes is the interquartile range that represents values between the 75th and 25th percentiles of individual Ct values. *p*-values are shown; Table S2: Median Ct values, SD, and median miRNA expression values in the plasma of patients with NSCLC (N = 72) and healthy volunteers (N = 33); Figure S2: Differential expression of miR-34a, miR-146a, miR-155, miR-200b, miR-200c, and miR-223 in the plasma of NSCLC patients (N = 69) treated with Nivolumab compared to healthy donors (N = 33); Figure S3: Correlation of miRNAs expression levels and patients’ clinicopathological characteristics. Correlation with (A) the histologic subtype, (B) bone metastasis, (C) liver metastasis, and (D) the presence of multiple metastatic sites. Expression levels of miRNAs were assessed by the 2^−∆ΔCt^ method, and U6 snRNA was used as a reference gene. Statistically significant differences were determined by the Mann-Whitney test, and the results are displayed on box plots. The horizontal line depicts the median, whereas the length of the boxes is the interquartile range that represents values between the 75th and 25th percentiles of individual fold change expression values. Relative expression values on the y-axis are plotted on a log10 scale. *p*-values are shown; Figure S4: Correlation of miRNAs expression levels and response to immunotherapy, (A) Correlation of miR-146a and PDDC in the SqCC subgroup (B) Correlation of miR-223 and PDDC in the SqCC subgroup, (C) Correlation of miR-34a and DCR in the Non-SqCC subgroup, (D) Correlation of miR-200c and ORR in the Non-SqCC subgroup and (E) Correlation of miR-200c and PDDC in the Non-SqCC subgroup. *p*-values are shown; Figure S5: Kaplan–Meier analysis for PFS (left column) and OS (right column) (A) miR-34a, (B) miR-146a, (C) miR-155, (D) miR-200b and (E) miR-223, based on the microRNA’s expression levels in the plasma of patients with NSCLC (N = 69). Median expression values for each microRNA subcategorized patients into high and low expression groups. Curves were compared using the log-rank test. *p*-values are shown; Figure S6: Kaplan-Meier analysis for PFS (left column) and OS (right column) based on the miRNAs’ expression levels in the plasma of patients with SqCC subtype (N = 31): (A) miR-34a, (B) miR-146a, (C) miR-155, (D) miR-200b, (E) miR-200c and (F) miR-223. Median expression values for each microRNA subcategorized patients into high and low expression groups. Curves were compared using the log-rank test. *p*-values are shown; Table S3: Univariate and Multivariate Cox regression analysis for Progression Free Survival (PFS) and Overall Survival (OS) in patients with SqCC subtype (N = 31) treated with Nivolumab; Figure S7: Kaplan–Meier analysis for PFS (left column) and OS (right column) based on the miRNAs’ expression levels in the plasma of patients with non-SqCC subtype (N = 38): (A) miR-146a, (B) miR-155, (C) miR-200b and (D) miR-223. Median expression values for each microRNA subcategorized patients into high and low expression groups. Curves were compared using the log-rank test. *p*-values are shown.

## Abbreviations

cDNA	Complementary DNA
CI	Confidence intervals
CT	Computed tomography
Ct	Cycle threshold
DCR	Disease Control Rate
ECOG	Eastern Cooperative Oncology Group
EDTA	Ethylenediaminetetraacetic acid
EMT	Epithelial to Mesenchymal Transition
HR	Hazard ratio
ICIs	Immune Checkpoint Inhibitors
MDSCs	Myeloid-Derived Suppressor Cells
miRNAs	microRNAs
MRI	Magnetic resonance imaging
NK	Natural killer cells
non-SqCC	non-Squamous
NSCLC	Non-Small Cell Lung Cancer
ORR	Objective Response Rate
OS	Overall survival
PD	Progression disease
PDDC	Prolonged Duration of Disease Control
PFS	Progression-free survival
PR	Partial response
PS	Performance status
RT-qPCR	Real-time quantitative polymerase chain reaction
SD	Stable disease
SqCC	Squamous
TILs	Tumor-infiltrating lymphocytes
TME	Tumor microenvironment
TNBC	Triple-negative breast cancer
Tregs	T regulatory cells
