# Supplementary material for: Plasma-Based microRNA Expression Analysis in Advanced Stage NSCLC Patients Treated with Nivolumab
Source: Cancers (Basel). 2022 Sep 28;14(19):4739. doi: 10.3390/cancers14194739 (PMC9564103; doi:10.3390/cancers14194739)
Supplement: Supplementary file 1 [file cancers-14-04739-s001.zip › cancers-1836980-supplementary.pdf]

Supplementary

Table S1. Assay ID for each miRNA used in the study.

Name	Assay ID
**hsa-miR-34a-5p**	000426
**hsa-miR-146a-5p**	000468
**hsa-miR-155-5p**	002623
**hsa-miR-200b-3p**	002251
**hsa-miR-200c-3p**	002300
**hsa-miR-223-3p**	002295
**U6 snRNA**	001973
**Cel-miR-39**	000200

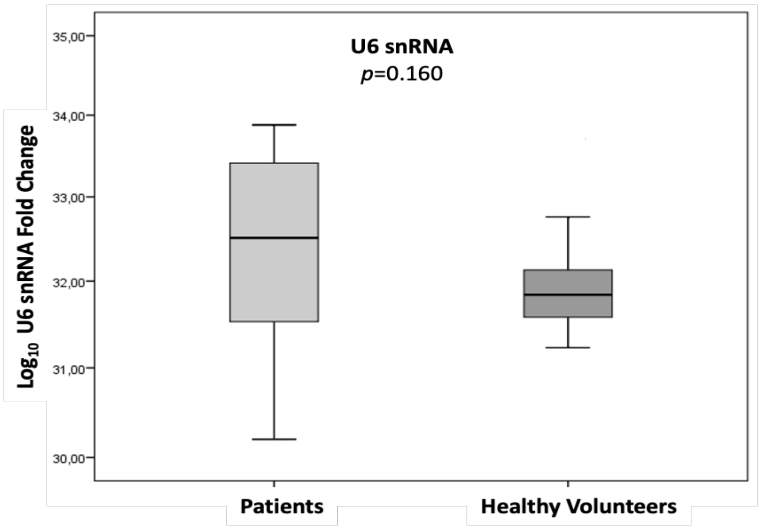

Figure S1. U6 snRNA expression levels between patientswith NSCLC and healthy donors. Mann-Whitney test was used to determine statistically significant differences and the results were displayed on box plots. Horizontal line depicts the median Ct value, whereas the length of the boxes is the interquartile range that represents values between the 75th and 25th percentiles of individual Ct values. *p* values are shown.

Table S2. Median Ct values, SD and median miRNA expression values in the plasma of patients with NSCLC (N=72) and healthy volunteers (N=33).

	NSCLC patients (N=69)			Healthy Volunteers (N=33)		*p* value
	Ct	SD	Median	Ct	SD	
**miR-34a**	32.76	1.56	1.42	34.55	1.15	$p<0.001$
**miR-146a**	26.07	1.36	110.97	28.50	0.99	$p<0.001$
**miR-155**	31.55	1.16	3.09	32.02	0.77	$p=0.646$
**miR-200b**	34.06	1.96	0.48	34.76	1.47	$p<0.001$
**miR-200c**	32.90	2.22	1.36	34.26	1.12	$p<0.001$
**miR-223**	21.94	1.35	1798.33	24.61	1.23	$p<0.001$

Ct, cycle threshold; SD, standard deviation; miRNA expression values were calculated by the 2-ΔCt method, Mann-Whitney test.

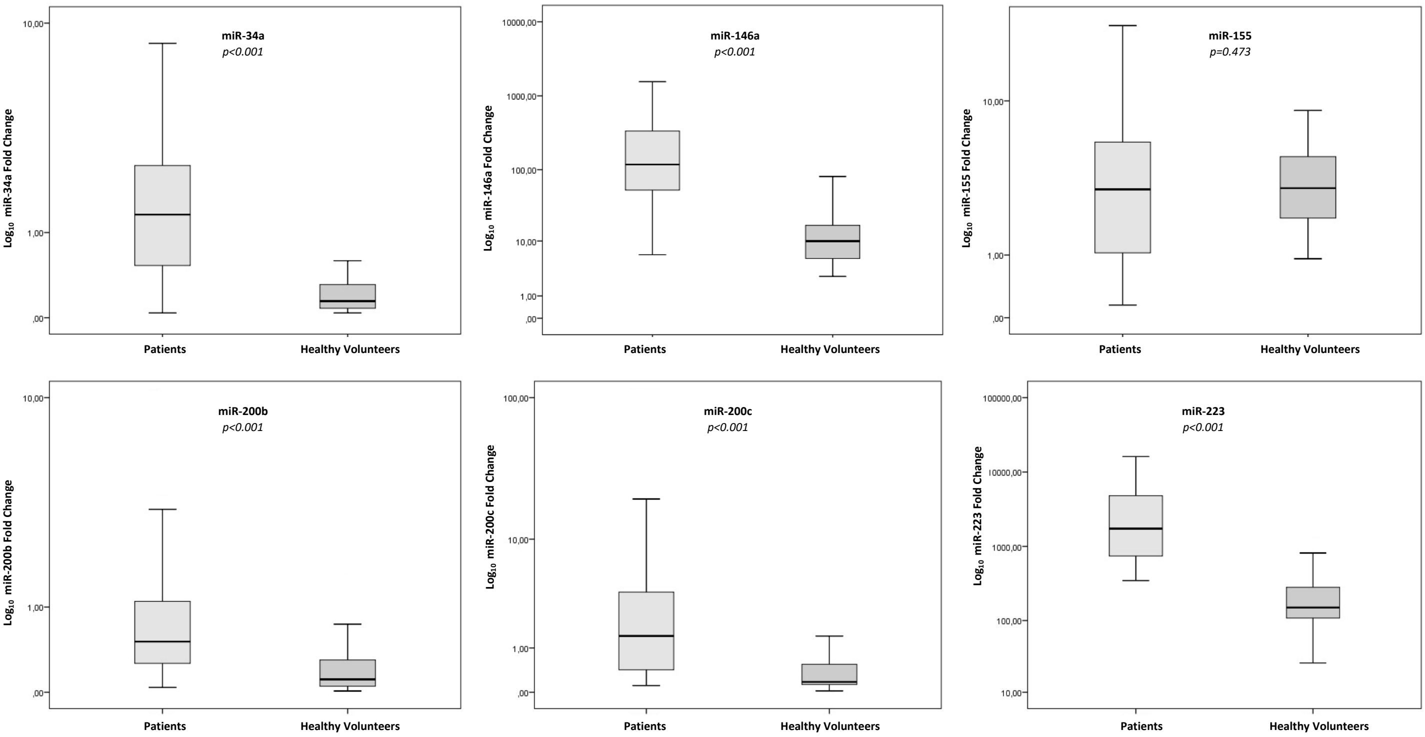

Figure S2. Differential expression of miR-34a, miR-146a, miR-155, miR-200b, miR-200c and miR-223 in the plasma of NSCLC patients (N=69) treated with Nivolumab compared to healthy donors (N=33).

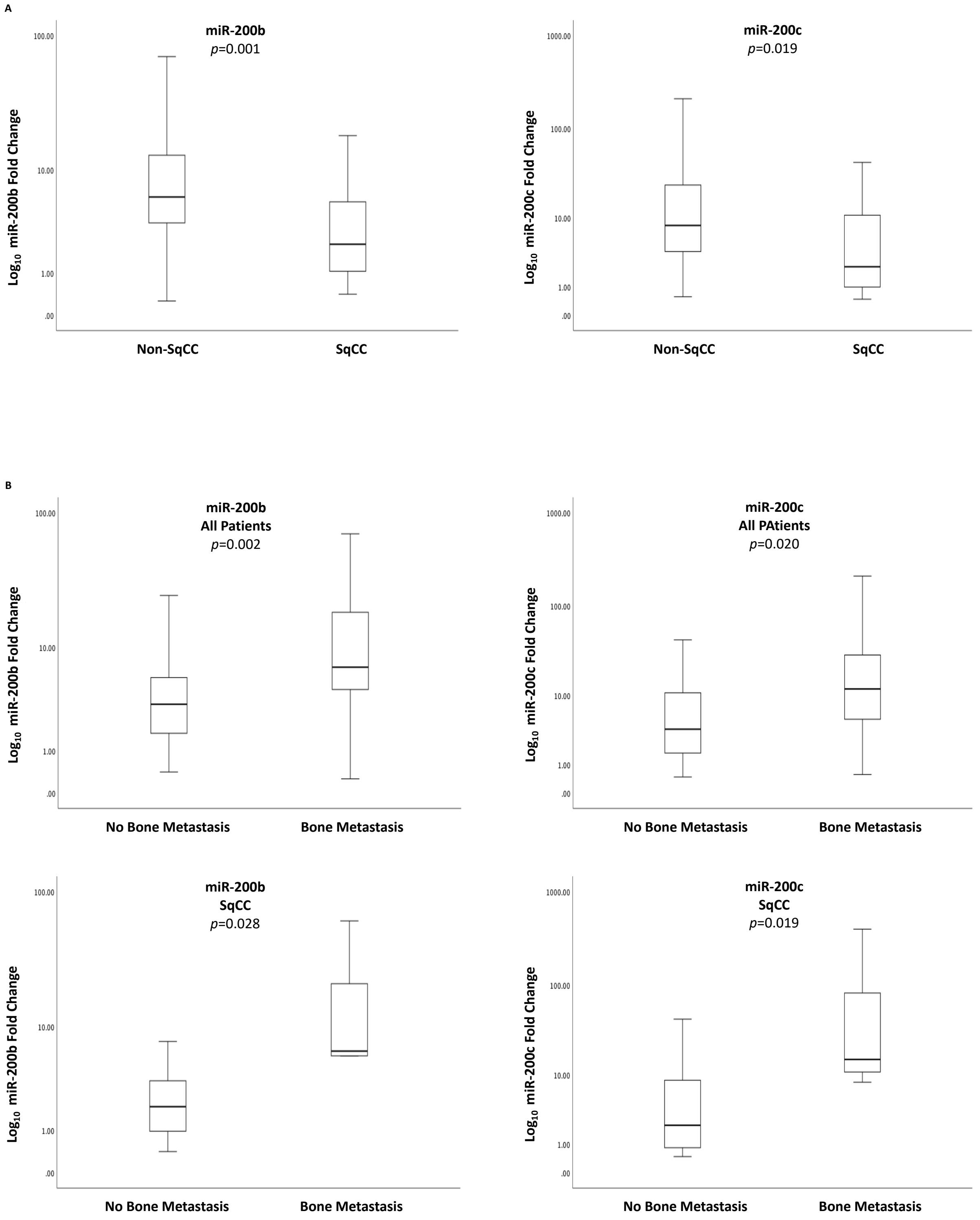
A
miR-200b
p=0.001
Log10 miR-200b Fold Change
100.00
10.00
1.00
.00
Non-SqCC
SqCC
miR-200c
p=0.019
Log10 miR-200c Fold Change
1000.00
100.00
10.00
1.00
.00
Non-SqCC
SqCC
B
miR-200b
All Patients
p=0.002
Log10 miR-200b Fold Change
No Bone Metastasis
Bone Metastasis
miR-200c
All PAtients
p=0.020
Log10 miR-200c Fold Change
No Bone Metastasis
Bone Metastasis
miR-200b
SqCC
p=0.028
Log10 miR-200b Fold Change
No Bone Metastasis
Bone Metastasis
miR-200c
SqCC
p=0.019
Log10 miR-200c Fold Change
No Bone Metastasis
Bone Metastasis

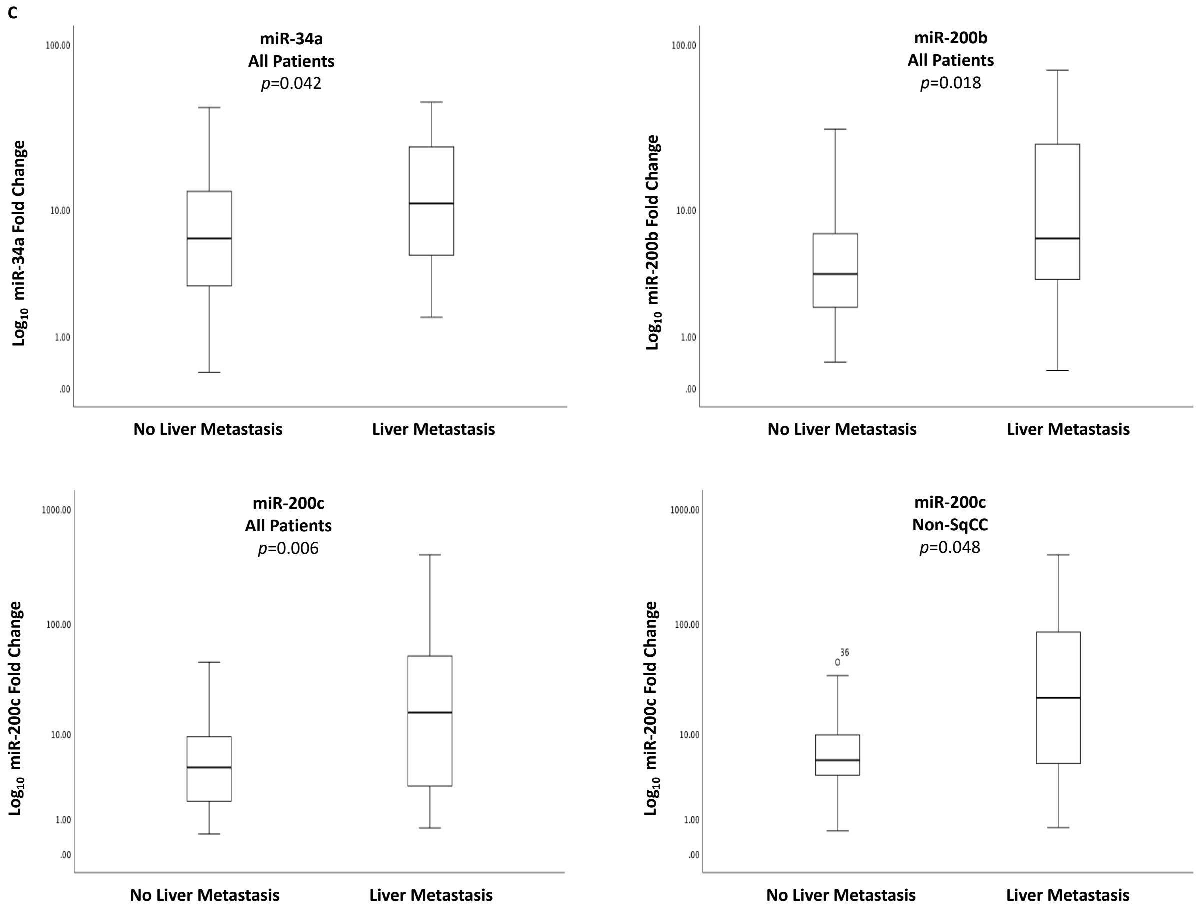

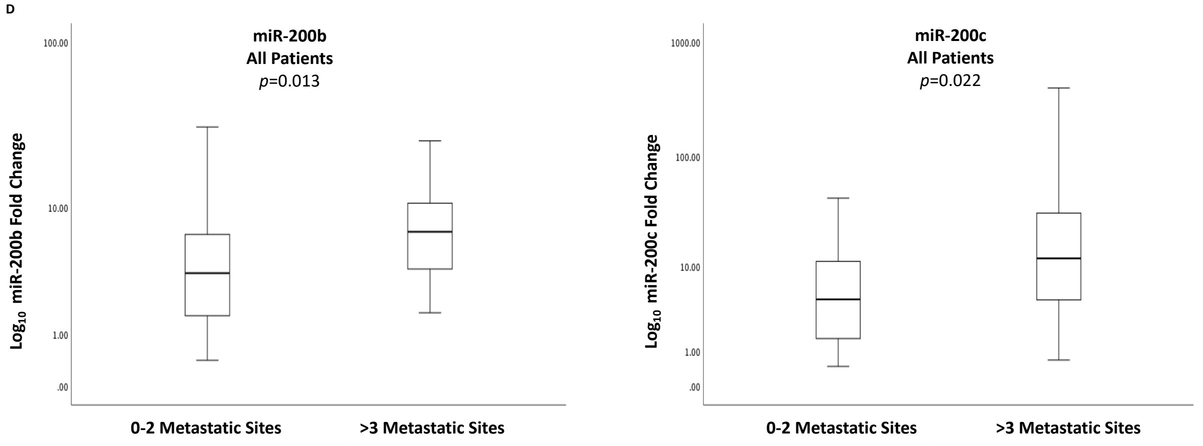

Figure S3. Correlation of miRNAs expression levels and patients' clinicopathological characteristics. Correlation with (A) the histologic subtype, (B) bone metastasis, (C) liver metastasis and (D) the presence of multiple metastatic sites. Expression levels of miRNAs were assessed by the $2^{-\Delta\Delta Ct}$ method and U6 snRNA was used as a reference gene. Statistically significant differences were determined by the Mann-Whitney test and the results are displayed on box plots. Horizontal line depicts median, whereas the length of the boxes is the interquartile range that represents values between the 75th and 25th percentiles of individual fold change expression values. Relative expression values on the *y*-axis are plotted on a $\log_{10}$ scale. *p* values are shown.

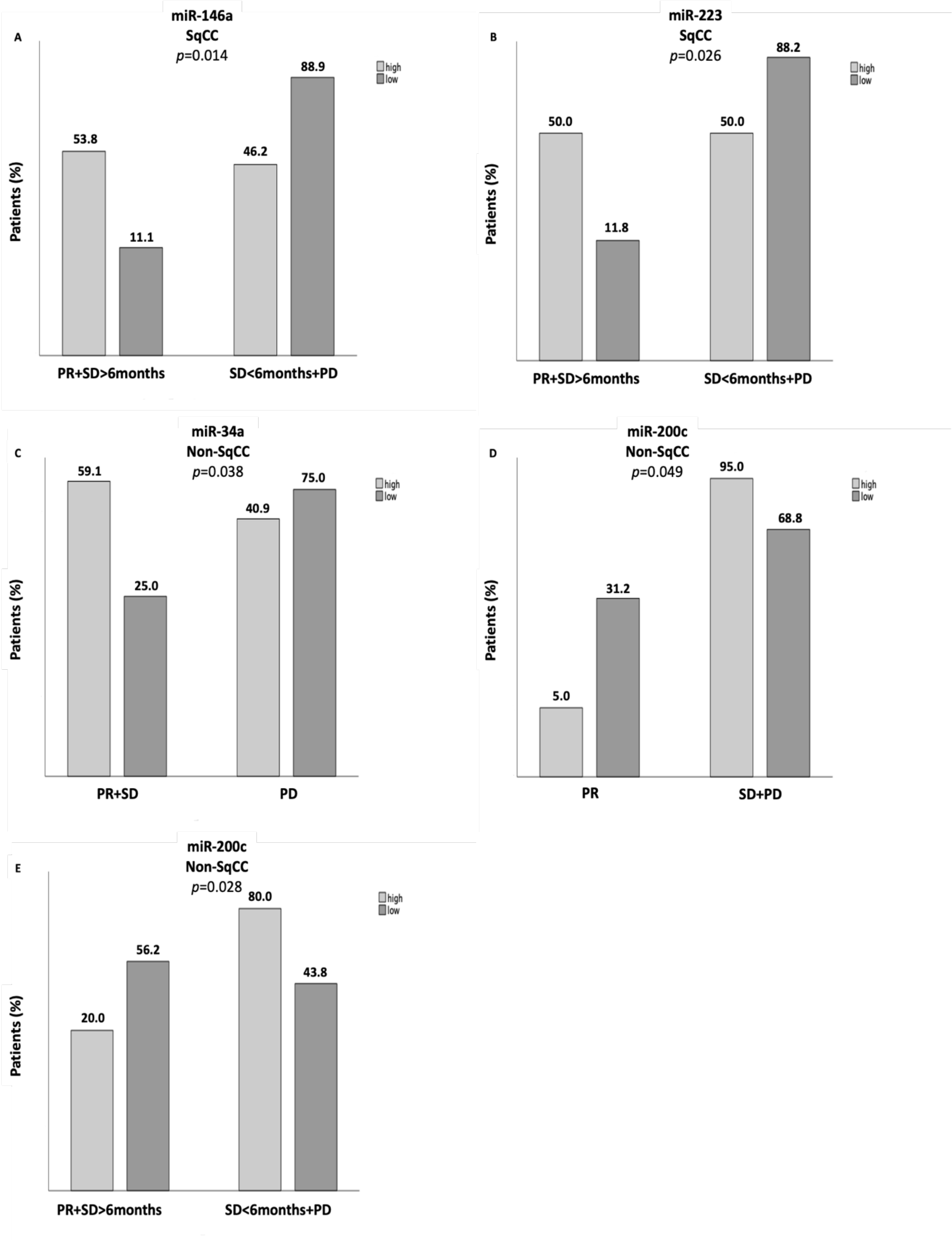

Figure S4. Correlation of miRNAs expression levels and response to immunotherapy, (A) Correlation of miR-146a and PDDC in the SqCC subgroup (B) Correlation of miR-223 and PDDC in the SqCC subgroup, (C) Correlation of miR-34a and DCR in the Non-SqCC subgroup, (D) Correlation of miR-200c and ORR in the Non-SqCC subgroup and (E) Correlation of miR-200c and PDDC in the Non-SqCC subgroup. p values are shown.

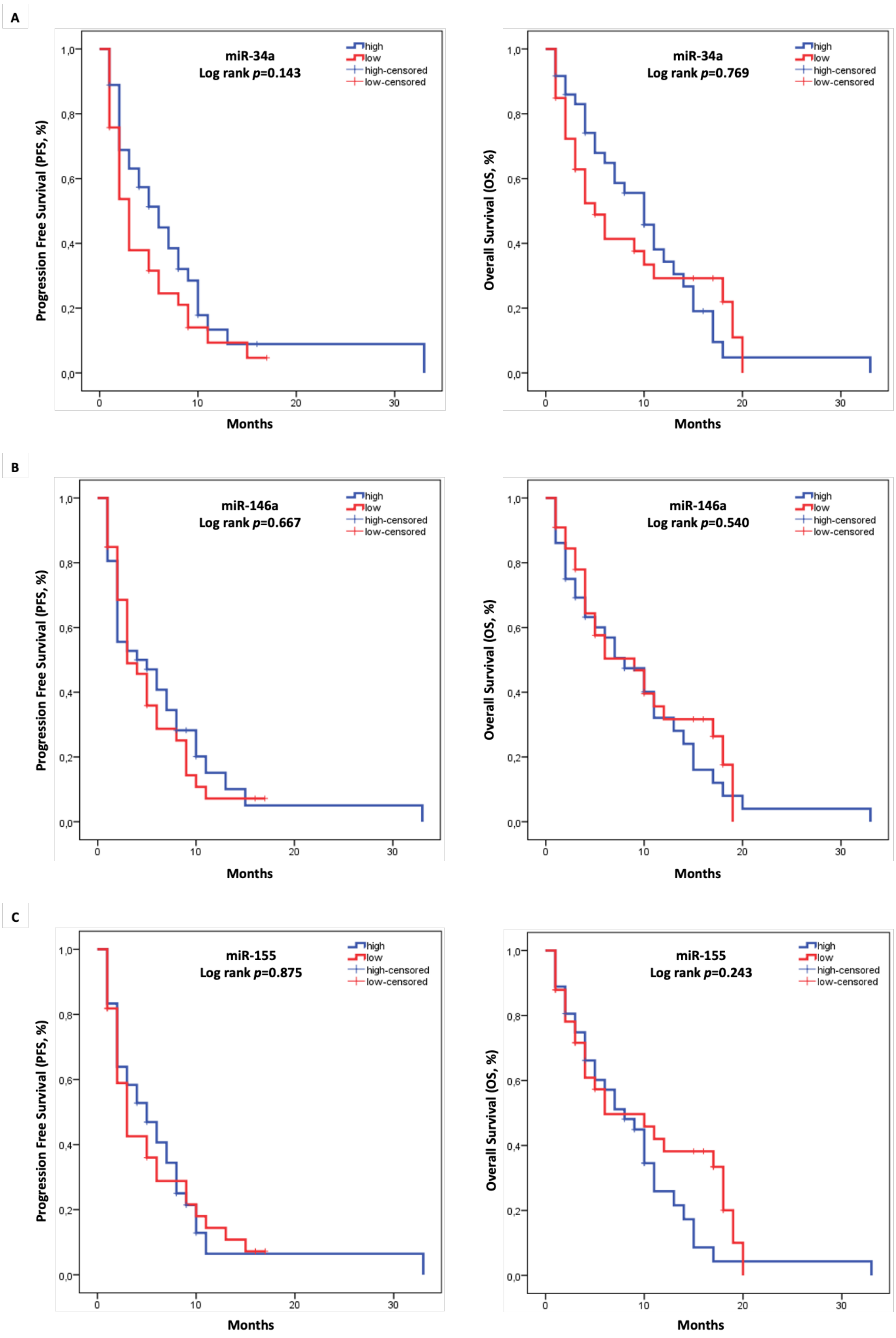
A
miR-34a
Log rank p=0.143
high
low
high-censored
low-censored
Progression Free Survival (PFS, %)
Months
miR-34a
Log rank p=0.769
Overall Survival (OS, %)
Months
B
miR-146a
Log rank p=0.667
Progression Free Survival (PFS, %)
Months
miR-146a
Log rank p=0.540
Overall Survival (OS, %)
Months
C
miR-155
Log rank p=0.875
Progression Free Survival (PFS, %)
Months
miR-155
Log rank p=0.243
Overall Survival (OS, %)
Months

D

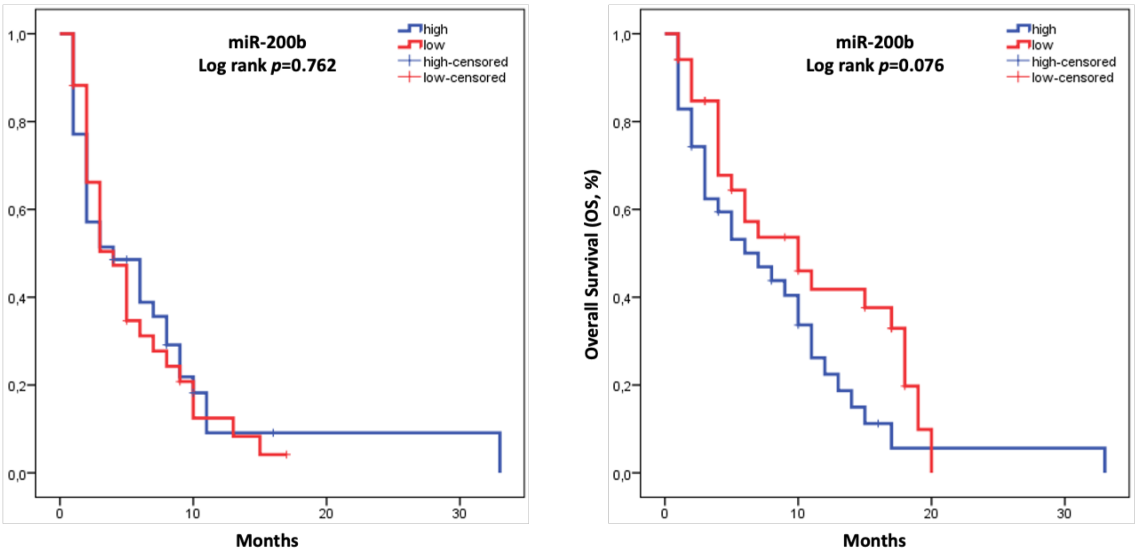

E

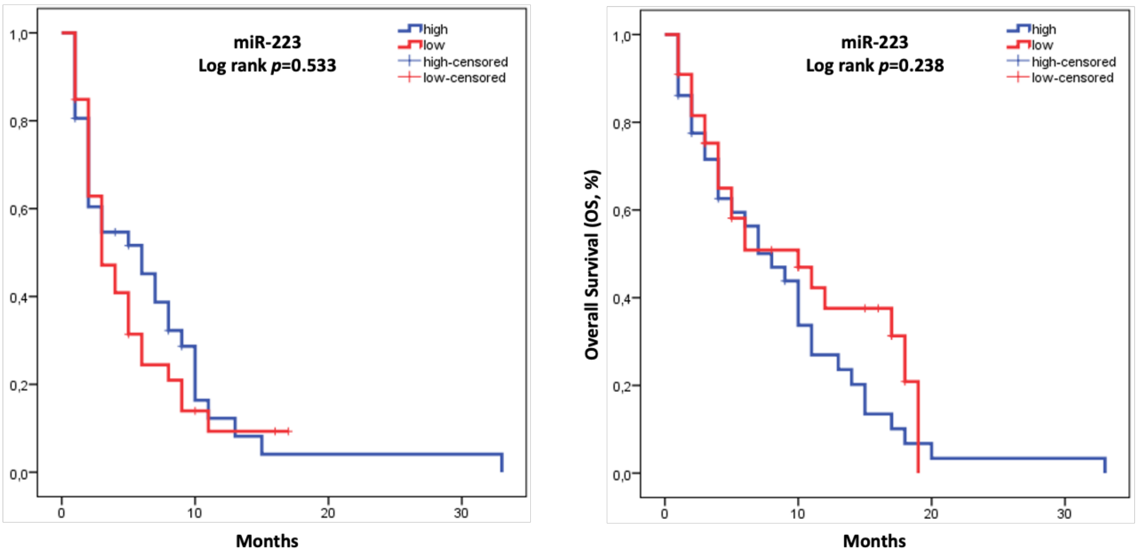

Figure S5. Kaplan-Meier analysis for PFS (left column) and OS (right column) (A) miR-34a, (B) miR-146a, (C) miR-155, (D) miR-200b and (E) miR-223, based on the microRNA's expression levels in the plasma of patients with NSCLC (N=69). Median expression values for each microRNA subcategorized patients into high and low expression groups. Curves were compared using the log rank test. *p* values are shown.

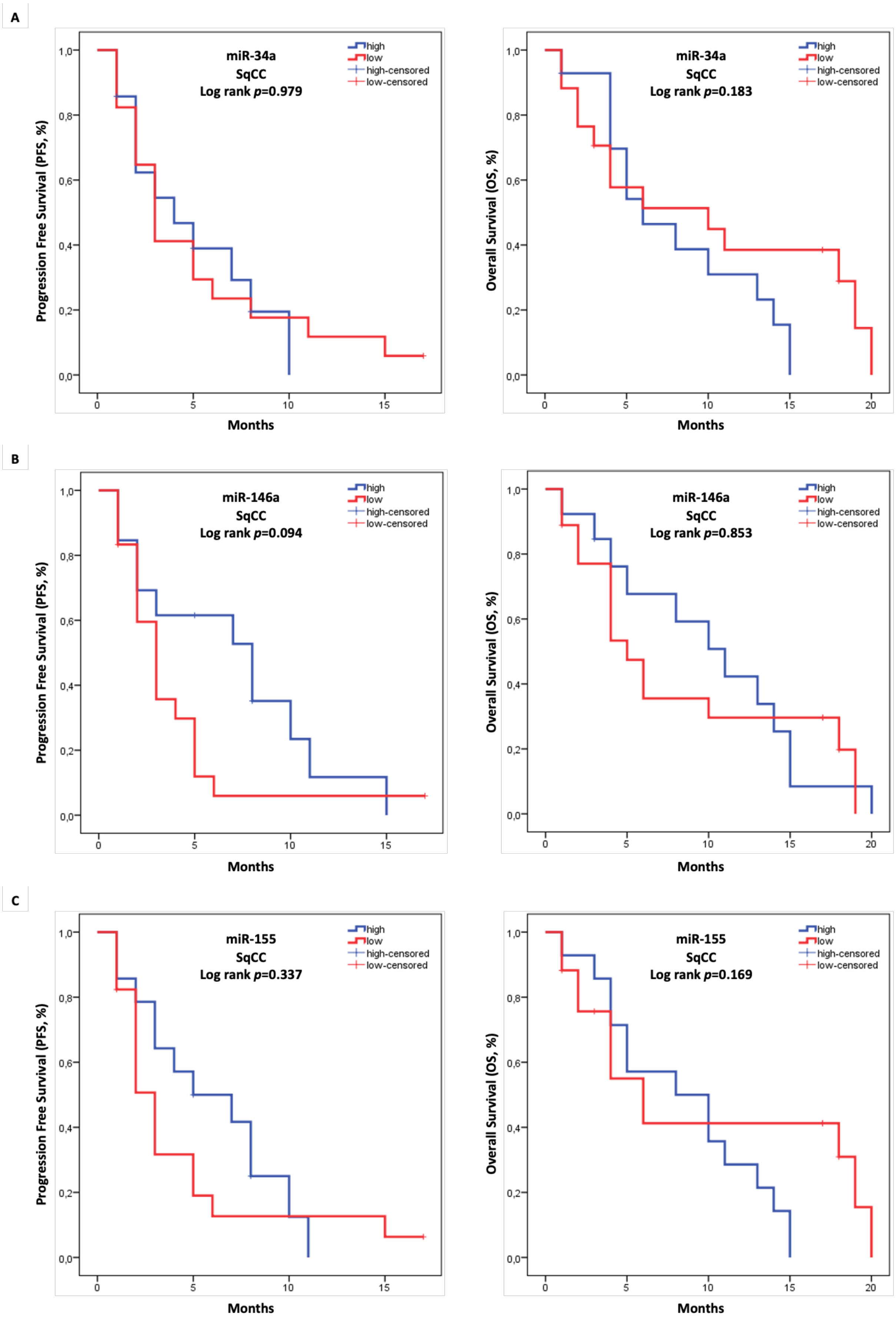

A
B
C
miR-34a
SqCC
Log rank p=0.979
Log rank p=0.183
miR-146a
Log rank p=0.094
Log rank p=0.853
miR-155
Log rank p=0.337
Log rank p=0.169
high
low
high-censored
low-censored
Progression Free Survival (PFS, %)
Overall Survival (OS, %)
Months

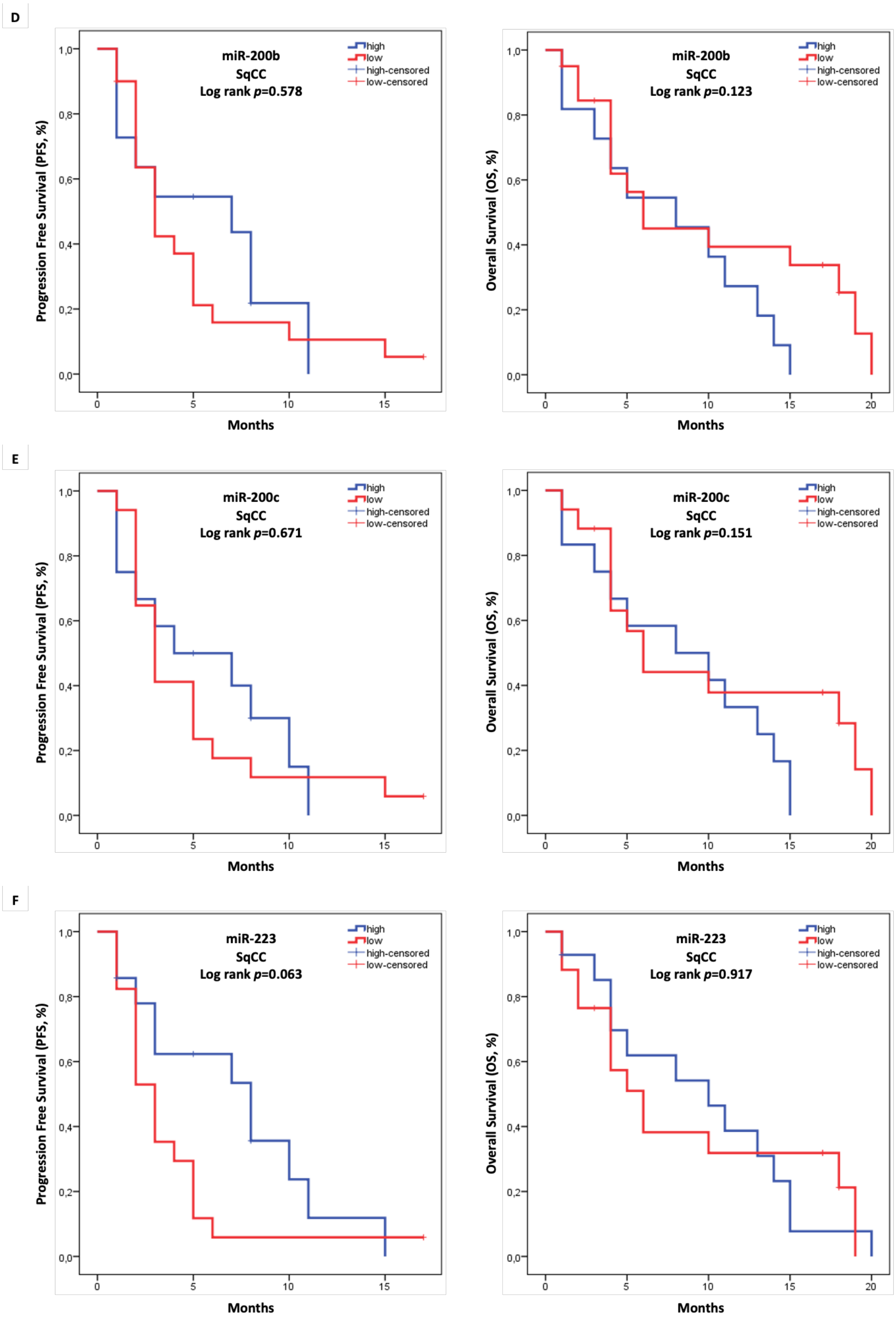

Figure S6. Kaplan-Meier analysis for PFS (left column) and OS (right column) based on the miRNAs' expression levels in the plasma of patients with SqCC subtype(N=31): (A) miR-34a, (B) miR-146a, (C) miR-155, (D) miR-200b,

(E) miR-200c and (F) miR-223. Median expression values for each microRNA subcategorized patients into high and low expression groups. Curves were compared using the log rank test. *p* values are shown.

Table S3. Univariate and Multivariate Cox regression analysis for Progression Free Survival (PFS) and Overall Survival (OS) in patients with SqCC subtype (N=31) treated with Nivolumab.

Variable	Progression Free Survival (PFS)				Overall Survival (OS)			
	Univariate Analysis		Multivariate Analysis		Univariate Analysis		Multivariate Analysis	
	HR (95% CI)	*p* Value	HR (95% CI)	*p* Value	HR (95% CI)	*p* Value	HR (95% CI)	*p* Value
Age (<60 vs. ≥60)	4.157 (0.892-19.381)	0.070	-	-	1.880 (0.249-14.182)	0.540	-	-
Gender (male vs. female)	1.540 (0.203-11.688)	0.676	-	-	5.497 (0.641-47.109)	0.120	-	-
Smoker (Yes vs. No)	1.540 (0.203-11.688)	0.676	-	-	5.497 (0.641-47.109)	0.120	-	-
ECOG PS (≥2 vs. 0-1)	1.894 (0.747-4.804)	0.178	-	-	4.267 (1.584-11.494)	0.004*	-	-
Immunotherapy Line (2nd vs. 3rd)	1.256 (0.294-5.377)	0.758	-	-	2.494 (0.335-18.584)	0.372	-	-
No. of Metastatic Sites (≥3 vs. 0-2)	1.232 (0.418-3.631)	0.706	-	-	2.227 (0.734-6.753)	0.157	-	-
miR-34a (low vs. high)	1.010 (0.457-2.231)	0.980	-	-	1.713 (0.743-3.952)	0.207	-	-
miR-146a (high vs. low)	1.883 (0.834-4.248)	0.128	-	-	1.075 (0.485-2.384)	0.859	-	-
miR-155 (high vs. low)	1.425 (0.648-3.131)	0.378	-	-	1.761 (0.751-4.127)	0.193	-	-
miR-200b (high vs. low)	1.240 (0.545-2.824)	0.608	-	-	1.859 (0.808-4.274)	0.145	-	-
miR-200c (high vs. low)	1.176 (0.523-2.642)	0.695	-	-	1.797 (0.771-4.189)	0.175	-	-
miR-223 (high vs. low)	2.006 (0.893-4.506)	0.092	-	-	1.041 (0.472-2.296)	0.921	-	-

HR, Hazard Ratio; CI, Confidence Intervals; ECOG PS, Eastern Cooperative Oncology Group Performance Status; patients classified into high and low expression groups according to the median value of each miRNA; Cox regression, * $p < 0.05$.

A

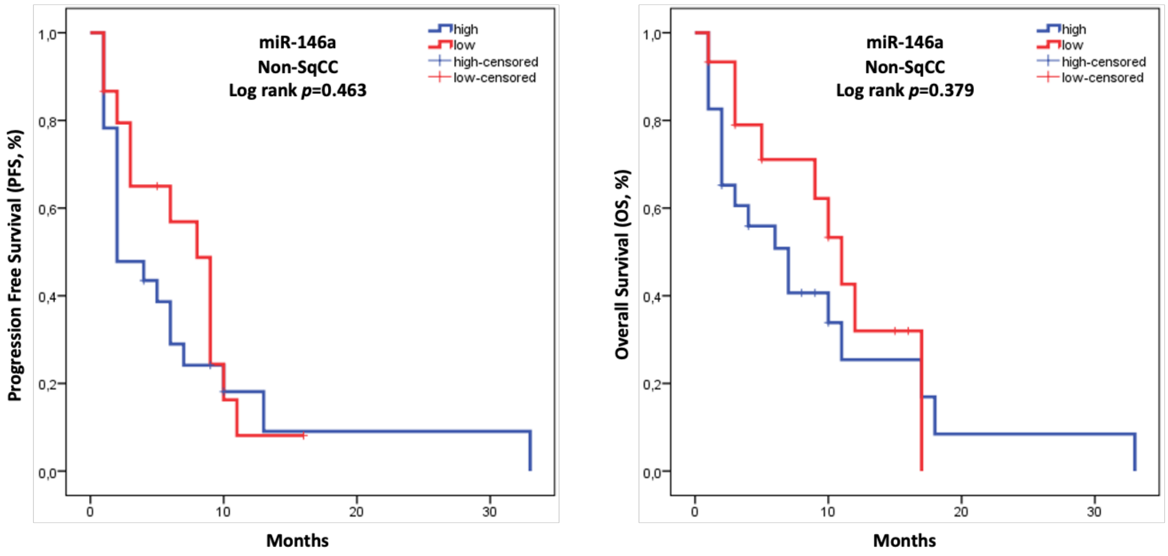
miR-146a
Non-SqCC
Log rank p=0.463
high
low
high-censored
low-censored
Progression Free Survival (PFS, %)
1,0
0,8
0,6
0,4
0,2
0,0
0
10
20
30
Months
miR-146a
Non-SqCC
Log rank p=0.379
high
low
high-censored
low-censored
Overall Survival (OS, %)
1,0
0,8
0,6
0,4
0,2
0,0
0
10
20
30
Months

B

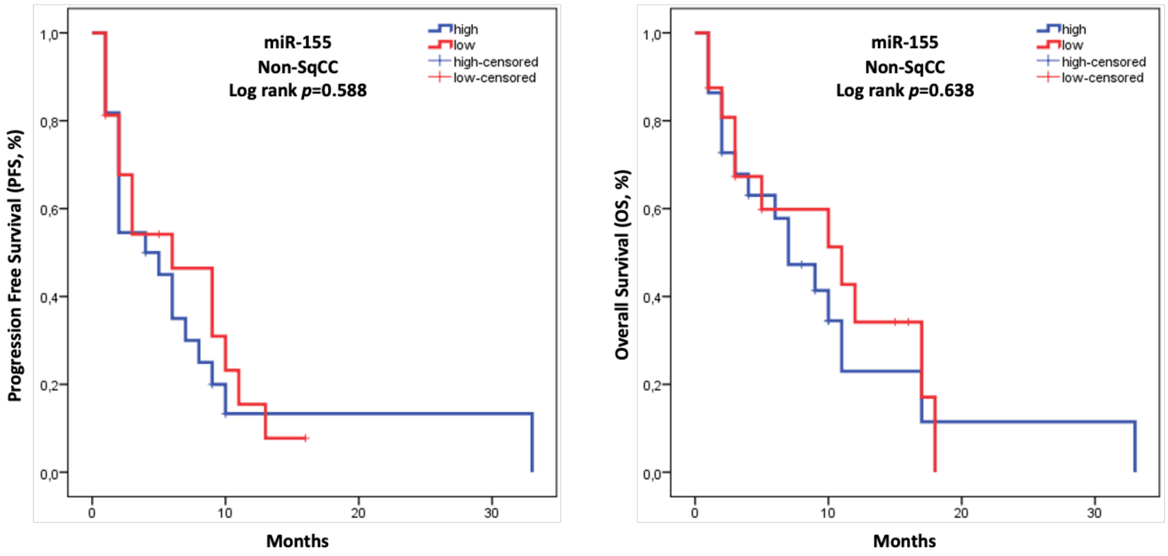
miR-155
Non-SqCC
Log rank p=0.588
high
low
high-censored
low-censored
Progression Free Survival (PFS, %)
1,0
0,8
0,6
0,4
0,2
0,0
0
10
20
30
Months
miR-155
Non-SqCC
Log rank p=0.638
high
low
high-censored
low-censored
Overall Survival (OS, %)
1,0
0,8
0,6
0,4
0,2
0,0
0
10
20
30
Months

C

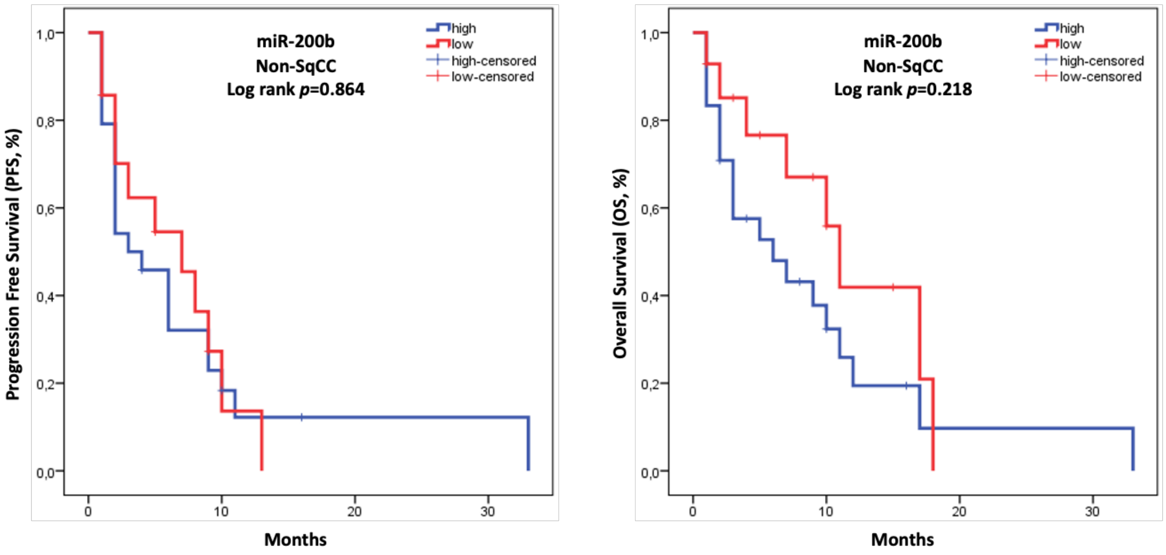

D

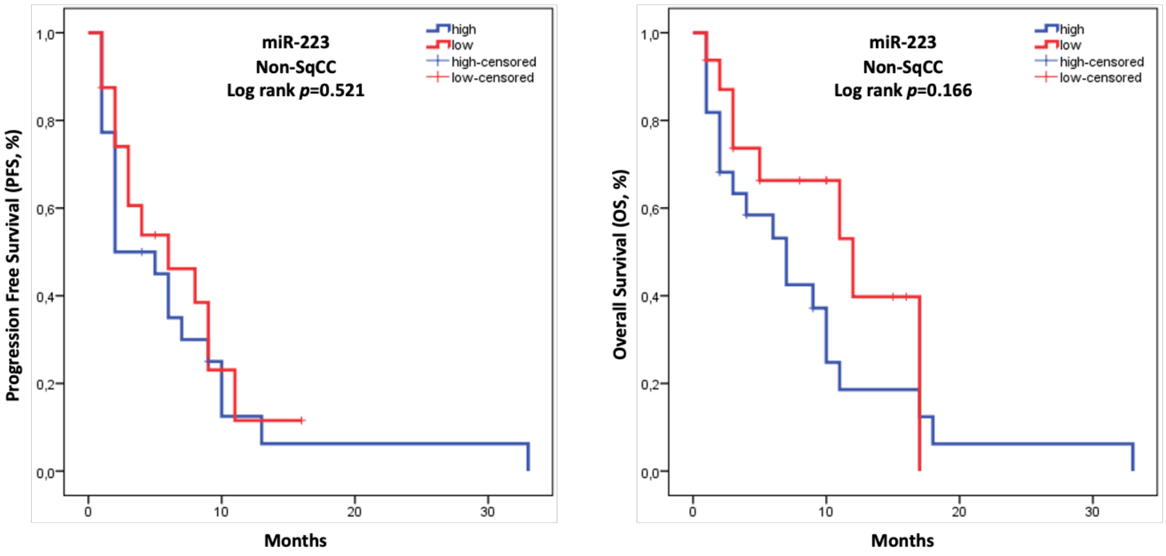

Figure S7. Kaplan-Meier analysis for PFS (left column) and OS (right column) based on the miRNAs' expression levels in the plasma of patients with non-SqCC subtype (N=38): (A) miR-146a, (B) miR-155, (C) miR-200b and (D) miR-223. Median expression values for each microRNA subcategorized patients into high and low expression groups. Curves were compared using the log rank test. *p* values are shown.
